# Vaccine development in *Staphylococcus aureus*: taking the biofilm phenotype into consideration

**DOI:** 10.1111/j.1574-695X.2010.00708.x

**Published:** 2010-06-29

**Authors:** Janette M Harro, Brian M Peters, Graeme A O'May, Nathan Archer, Patrick Kerns, Ranjani Prabhakara, Mark E Shirtliff

**Affiliations:** 1 Department of Microbial Pathogenesis, Dental School, University of MarylandBaltimore, MD, USA; 2 Graduate Program in Life Sciences, Microbiology and Immunology Program, School of Medicine, University of MarylandBaltimore, MD, USA; 3 Department of Microbiology and Immunology, School of Medicine, University of MarylandBaltimore, MD, USA

**Keywords:** *Staphylococcus aureus*, vaccine, biofilm

## Abstract

Vaccine development against pathogenic bacteria is an imperative initiative as bacteria are gaining resistance to current antimicrobial therapies and few novel antibiotics are being developed. Candidate antigens for vaccine development can be identified by a multitude of high-throughput technologies that were accelerated by access to complete genomes. While considerable success has been achieved in vaccine development against bacterial pathogens, many species with multiple virulence factors and modes of infection have provided reasonable challenges in identifying protective antigens. In particular, vaccine candidates should be evaluated in the context of the complex disease properties, whether planktonic (e.g. sepsis and pneumonia) and/or biofilm associated (e.g. indwelling medical device infections). Because of the phenotypic differences between these modes of growth, those vaccine candidates chosen only for their efficacy in one disease state may fail against other infections. This review will summarize the history and types of bacterial vaccines and adjuvants as well as present an overview of modern antigen discovery and complications brought about by polymicrobial infections. Finally, we will also use one of the better studied microbial species that uses differential, multifactorial protein profiles to mediate an array of diseases, *Staphylococcus aureus*, to outline some of the more recently identified problematic issues in vaccine development in this biofilm-forming species.

## A history of bacterial vaccines

The first bacterial vaccines developed used whole bacteria in either a live, attenuated vaccine (LAV) or a killed, whole-cell vaccine (KWCV). LAVs are generated either by repeat passage of the pathogen in a nonstandard host or in culture media or more recently by the targeted deletion of gene(s) that enable a pathogenic phenotype in humans. Louis Pasteur's work on the chicken cholera bacterium (*Pasteurella multocida*) and anthrax are the earliest examples of bacterial LAVs. Subsequent research on bacterial LAVs led to the development of the BCG vaccine for tuberculosis (Bastos *et al.*, 2009), the salmonella Ty21a vaccine for the prevention of typhoid (Wahdan *et al.*, 1980), and the CVD103-Hgr vaccine against cholera (Ketley *et al.*, 1993; Levine & Kaper, 1993;). These vaccines continue to be used in developed and developing countries, because LAVs often confer a robust, long-lasting protection without the need to administer frequent booster shots.

Salmon and Smith subsequently laid the foundation for administering a heat-killed suspension of bacteria and paved the way for KWCVs. These vaccines were easy to produce, but had frequent adverse effects such as fever, anorexia, and swelling or induration induced by lipopolysaccharide. These drawbacks have led to almost complete clinical disuse of KWCVs in the United States. In response to these side effects, acellular, protein versions of traditional vaccines such as the acellular pertussis vaccines (Decker & Edwards, 2000) and the acellular anthrax vaccines (Friedlander & Little, 2009) followed. Rationales for immunizing with a limited number of antigens are reduced reactogenicity and avoidance of autoimmunity resulting from molecular mimicry by bacterial antigens (Zorzeto *et al.*, 2009). A limitation is that immunity elicited by a single antigen wanes more quickly than that generated by a LAV.

Alternatively, the tetanus and diphtheria toxoid vaccines developed in the 1920s are currently being used with minor alterations to their manufacture (Plotkin *et al.*, 2008). The toxoid vaccine lacks the toxin's pathogenic qualities and is used for vaccination to generate neutralizing antibodies against the toxin. Because single toxins are responsible for the bulk of *Clostridium tetani* and *Corynebacterium diphtheriae* pathogenesis, a robust immunoglobulin G (IgG) neutralizing antibody response that targets and blocks the toxin interrupts the disease process.

A better understanding of the critical role of polysaccharide capsules in the pathogenesis of *Streptococcus pneumoniae* and *Haemophilus influenzae* led to the development of polysaccharide vaccines (PSVs) against these pathogens (Riley *et al.*, 1977; Robbins *et al.*, 1983; Mufson *et al.*, 1985;) as well as a PSV against *Neisseria meningitidis* serotypes A, C, W-135, and Y (Artenstein *et al.*, 1970; Armand *et al.*, 1982; Ambrosch *et al.*, 1983;). Because of suboptimal immunogenicity elicited by polysaccharide, PSVs are being eliminated and replaced by polysaccharide–protein conjugate vaccines. Conjugate vaccines elicit a robust IgG response imparted by the protein carrier, which converts the polysaccharide from a T-cell-independent immunogen into a T-cell-dependent immunogen (Perez-Melgosa *et al.*, 2001).

Innovations to vaccine design over the years have resulted in a number of successful bacterial vaccines that supplant earlier, less effective vaccines. Currently, several competing cholera (Lopez *et al.*, 2008) and typhoid vaccines (Fraser *et al.*, 2007) are available. A closer examination of these vaccines defines the pros and cons of certain vaccine strategies (Table 1).

**Table 1:** General characteristics of classical bacterial vaccine types

Vaccine type	Pros	Cons
Killed, whole bacteria	Relatively simple to make Produces a protective immune response for many organisms	Highly reactogenic in many cases, this has rendered vaccines unusable or unpopular Risk of induction of autoimmunity via molecular mimicry Booster doses often needed
Live, attenuated bacteria	More robust and longer lasting immunity relative to killed, whole bacteria	Possibility of disease in immunocompromised patients Possibility of reacquisition of lost virulence resulting in disease Risk of secondary transmission
Toxoid	Excellent at generating toxin neutralizing antibodies Markedly less reactogenic compared with killed, whole bacteria	Multiple doses often needed Epitope must be highly conserved
Protein only	Markedly less reactogenic compared with killed, whole bacteria	Multiple doses often needed Epitope must be highly conserved
Polysaccharide only	Markedly less reactogenic compared with killed, whole bacteria	Multiple doses often needed Epitope must be highly conserved
Polysaccharide–protein conjugate	Improved antibody titers relative to polysaccharide only Decreased carriage for meningococcal and pneumococcal vaccines Can generate longer lasting immunity relative to polysaccharide vaccines Markedly less reactogenic compared with killed, whole bacteria	Meningococcal conjugate vaccine not currently recommended for children under age 11

Although vaccinology has made significant progress (Table 2), many challenges remain to date. When dealing with bacterial pathogens that can cause multiple forms of diseases through a large number of virulence factors, often traded between individual strains and species by horizontal gene transfer, protection via a single component vaccine is likely to be elusive. *Staphylococcus aureus* is an example of such a pathogen. This microbial species has dozens of known toxins, multiple immunoavoidance, and adherence factors, most of which demonstrate transient, timed, and disease-specific expression (DeLeo *et al.*, 2009). Therefore, a successful vaccine will likely be required to provide protective antibody titers against multiple antigens (Zecconi *et al.*, 2005).

**Table 2:** Common bacterial vaccines

Pathogen (disease)	Vaccine type	Composition	Current status
*Bacillus anthracis* (anthrax)	Live, attenuated	Sterne live-attenuated strains	Not available in the United States for humans, only for veterinary use
Acellular		Cell-free culture supernatant adsorbed to aluminum hydroxide; believed to contain mostly the protective antigen of the anthrax toxins	Not available to the public in the United States
*Bordetella pertussis* (pertussis)	Killed, whole cell	Killed pathogenic bacteria	Completely replaced by acellular vaccine in the United States and many developed countries
Acellular		Inactivated pertussis toxin plus one or more of the following proteins: hemaglutinin, pertactin, or fimbriae types 2 and 3	Approved for clinical use in the United States
*Borrelia burgdorferi* (Lyme disease)	Killed, whole cell	Inactivated whole-cell vaccine with proprietary polymer adjuvant or bivalent whole-cell killed	Veterinary vaccines for dogs
Lipoprotein		Lyme OspA recombinant lipoprotein	Withdrawn from clinical use in 2002
*Clostridium tetani* (tetanus)	Toxoid	Formaldehyde detoxified tetanus toxin	Currently licensed in the United States in several combinations
*Corynebacterium diphtheriae* (diphtheria)	Toxoid	Diphtheria toxoid adsorbed to aluminum salt	Currently licensed in the United States in several combinations
*Coxiella burnetii* (Q fever)	Killed, whole cell	Killed *C. burnetii*	Not commercially available in the United States
*Haemophilus influenzae* type B (pneumonia and meningitis)	Polysaccharide	Polyribosylribitol phosphate (PRP)	Not effective in children younger than 18 months (the population that experiences the most severe disease), not currently used in the United States
	Polysaccharide–protein conjugate	PRP or HbOC linked to either diphtheria toxoid or the outer membrane protein complex of *N. meningitidis*	Four currently licensed conjugate vaccines in the United States
*Mycobacterium tuberculosis* (tuberculosis)	Live, attenuated	Bacille Calmette-Geurin (BCG)	Widespread global use; rarely administered in the United States
*Neisseria meningitidis* (meningitis)	Polysaccharide–protein conjugate	Quadrivalent vs. A, C, Y, and W-135 strains	Currently licensed in the United States
*Rickettsia rickettsii* (typhus)	Killed, whole cell	Inactivated chick embryo cultured *R. rickettsii*	No currently licensed vaccine in the United States
*Salmonella typhi* (Typhoid)	Killed, whole cell	Heat- and phenol-inactivated *S. typhi*	No longer available in the United States
	Killed, whole cell	Acetone inactivated parenteral vaccine	Only available to the United States Armed Forces
	Live, attenuated	Ty21a galactose nonfermenting *S. typhi*	Available in the United States
	Polysaccharide	Vi capsular antigen	Available in the United States
	Polysaccharide–protein conjugate (Vi-rEPA)	Vi capsular antigen conjugated to *Pseudomonas aeruginosa* recombinant exotoxin A	In development
*Streptococcus pneumoniae* (pneumonia and meningitis)	Killed, whole cell	Monovalent killed	Abandoned, not available
	Polysaccharide	6-, 14-, and 23-valent polysaccharide vaccines	No longer used in the United States because it couldn't be used for children <2 years old and superior protection was afforded by conjugate vaccines
	Polysaccharide–protein conjugate	7-valent polysaccharide conjugated to diphtheria CRM_197_ carrier protein	Currently licensed for prevention of infant and child meningitis
	Polysaccharide	23-valent polysaccharide	Licensed for the prevention of pneumonia in patients of 65 years of age or older or immunosuppressed patients over the age of two
*Vibrio cholerae* (Cholera)	Killed, whole cell	Killed pathogenic bacteria	Licensed, but not widely used
	Killed, whole cell plus recombinant protein (WC-rBS)	Two heat-killed strains of *V. cholerae* plus recombinant cholera toxin B	Only approved for experimental use in the United States
	Live, attenuated (CVD103-Hgr)	Pathogenic bacteria with the cholera toxin B subunit deleted	Only approved for experimental use in the United States
*Yersinia pestis* (Plague)	Killed, whole cell (Haffkine vaccine)	Heat-inactivated whole organism	Generated severe AE's, never widely adopted
	Killed, whole cell	Formalin-inactivated *Y. pestis*	Formerly licensed for sale and used in military personnel during Vietnam War; no longer available due to marked AE's to initial and booster doses

AE, adverse event; HbOC, *Haemophilus* b oligosaccharide conjugate (derivative of PRP); PRP, polyribosylribitol phosphate.

## Types and modes of delivery of vaccines

Recombinant subunit protein technology has become the main strategy in the development of vaccines against infectious diseases. Subunit vaccines offer several advantages over previous vaccine strategies. Recombinant subunit vaccines are safe or less reactogenic with a defined composition, which is due to its genetic-based approach and antigen expression in nonpathogenic bacterial strains. Other advantages include multiple modes of delivery and further engineering of the subunit (Liljeqvist & Stahl, 1999; Hansson *et al.*, 2000;). The main drawbacks of subunit vaccines are the requirement of an adjuvant and multiple doses as well as low immunogenicity and a short half-life, which can be improved by conjugating the protein subunit to another protein or molecule (Hudecz, 2001; Tugyi *et al.*, 2008;). Conjugation of an antibody, adhesion factor, or other molecule (such as cholera toxin B subunit) to the peptide can target it to immunologically relevant sites or cells to improve response. Recombinant subunit vaccine efficacy is also reliant on the route of administration.

Current delivery methods include parenteral (e.g. transcutaneous and intramuscular) and mucosal (e.g. intranasal and oral) vaccines. The skin serves as a functional barrier by preventing harmful molecules and organisms from invading the host. Langerhans cells, a class of antigen-presenting cells, present antigens in the epidermal layer and the accessibility of the skin makes parenteral vaccination a favorable delivery method. The parenteral route of vaccine delivery is an effective inducer of systemic immunity represented by significant serum IgG titers and cytokine expression in lymph nodes. Nevertheless, this mode of vaccine delivery is deficient in its ability to initiate a mucosal immune response.

The mucosal surface is resident to the majority of lymphocytes found in the human body and is also the main entry point for infectious agents. This makes targeting vaccines to the mucosal sites crucial for immunity. The main advantage of mucosal vaccination over parenteral is the induction of IgA secretion at mucosal sites in combination with systemic IgG titers. Secreted IgA prevents the colonization and invasion of pathogens and neutralizes toxins at the mucosa (Slutter *et al.*, 2008). Mucosal vaccination leads to antigen-specific B cell memory, with the caveat that a proper immunostimulating compound is used (Vajdy, 2006). Antigen delivered without an adjuvant leads to mucosal tolerance, resulting in clonal deletion or induction of anergy of antigen-specific lymphocytes (Ogra *et al.*, 2001). In addition to mucosal tolerance, inefficient uptake of antigen and delivery to antigen-presenting cells is another disadvantage of mucosal vaccination (Slutter *et al.*, 2008). Mucosal vaccination has the potential to alleviate the innumerable diseases caused by pathogenic bacteria, viruses, and parasites by providing complete protection through IgA-mediated mucosal and IgG-mediated systemic immunity. Overcoming the hurdles of mucosal tolerance and inefficient antigen delivery may augment the vaccines currently in clinical trials.

## Adjuvants

Adjuvants work by stimulating the innate immune response, which is a required step in activating adaptive immunity. Cytokines and chemokines expressed upon stimulation of the innate immune response attract leukocytes to the local environment and cause maturation of antigen-presenting cells such as dendritic cells (DCs). The resident DCs are effective messengers between the innate and the adaptive response due to their enhanced antigen-presenting capabilities and ability to become polarized. Adjuvants promote cytokine expression within a microenvironment that polarizes DCs to mediate the expression of Th1 or Th2 cytokines and costimulatory molecules. In the draining lymph nodes, polarized DCs present the antigen to naïve T-cells. The development of Th0 to Th1, Th2, or other T-helper cells during antigen presentation is dependent on the expression of polarizing cytokines and costimulatory receptors produced by DCs. T-cells activated during this process potentiate the subsequent adaptive immune response.

Selecting the appropriate adjuvants for vaccine development is crucial, because they play a critical role in the development and polarization of the adaptive immune response. Adjuvants have been found to favor either a Th1 or a Th2 response, suggesting the production of Th1- and Th2-polarizing cytokines at the site of administration. To understand the immune response initiated by an adjuvant, whether it be Th1 or Th2, becomes essential in the selection of an adjuvant for vaccine design. Few adjuvants exist in the clinical realm; however, many are being tested experimentally. Table 3 details supplemental information on the current and experimental adjuvants.

**Table 3:** Adjuvant-dependent effector T cell differentiation

Adjuvants	Clinical status	Immune response	Experimental observations to designate immune response		References
Alum	Only one approved for US vaccines	TH2	TH1	No IgG2a titer No IFN-γ	Uddowla *et al.* (2007)Brewer (2006)
			TH2	High IgG1 titer IL-4 and IL-5 produced	Uddowla *et al.* (2007)Brewer (2006)
MF59	Fluad influenza vaccine*	TH2	TH1	Low IgG2a titer	Valensi *et al.* (1994), Wack *et al.* (2008)
			TH2	High IgG1 IL-5, IL-4, and THF-α produced	Valensi *et al.* (1994), Wack *et al.* (2008)
MF59 with CpG	No clinical application†	TH1	TH1	High IgG2a titer IFN-γ produced	Wack *et al.* (2008)
			TH2	Low IgG1 titer IL-5 suppressed	Wack *et al.* (2008)
AS04	Cervarix* (HPV)–Fendrix* (Hepatitis B)	TH1	TH1	High IgG2a IL-2 and IFN-γ produced	Korsholm *et al.* (2010), Didierlaurent *et al.* (2009)
			TH2	Low IgG1 IL-6 and THF-α produced	Korsholm *et al.* (2010), Didierlaurent *et al.* (2009)
c-di-GMP	No clinical application†	TH1/TH2	TH1	High IgG2a and IgG2b IFN-γ, THF-α, IL-12, MCP-1, and RANTES produced	Karaolis *et al.* (2007), Hu *et al.* (2009)
			TH2	High IgG1 and IgG3	Hu *et al.* (2009)

* European-approved vaccine application only.

† Not approved for human vaccine applications.

Adjuvants are potent inducers of innate immunity. They are often needed for an effective and protective adaptive immune response against pathogens. The Th response stimulated by vaccination is dependent on the cytokine milieu produced locally by an adjuvant, and the resultant polarization of antigen-presenting cells. Also, planktonic vs. biofilm-mediated diseases initiated by the same pathogen complicate vaccine development as each phenotype may require different Th responses to provide postvaccination protection. Research on the immunostimulating properties of molecules will elucidate future adjuvants and provide even greater options for vaccine development.

## Novel strategies for antigen selection: highlighting *S. aureus* advances

Vaccine design changed dramatically with advancements in genome sequencing technologies that enable rapid completion of genomes. Since the publication of the *H. influenzae* genome in 1995, the NCBI genome project reports that 1026 complete microbial genomes have been published including ones for 15 *S. aureus* strains (Fleischmann *et al.*, 1995) (http://www.ncbi.nlm.nih.gov/genomeprj). Access to complete genomes and bioinformatic technologies to manage and analyze the data has advanced high-throughput molecular techniques for genomic, transcriptomic, and proteomic analyses of microbial growth and pathogenesis (Kaushik & Sehgal, 2008; Zagursky & Anderson, 2008;). Genome-based technologies provide rapid identification of vaccine candidates compared with the conventional vaccine approaches, which identify and analyze individual virulence factors from pathogens grown *in vitro* (Rappuoli, 2000). Vaccines developed via genome-based technologies will still slowly transition into clinical phases after rapid identification, because these vaccines require the same rigorous evaluations using *in vitro* assays and animal models to validate functional activity as conventionally derived vaccines. As this review focuses on vaccine development against *S. aureus* to highlight *in vivo* phenotypes (e.g. biofilm formation and polymicrobial infection) that should be considered during antigen identification, we choose to present genome-based strategies and other technologies that identified putative *S. aureus* virulence factors and/or vaccine candidates. Vaccines comprised of antigenic candidates identified by these strategies may provide protection against *S. aureus* infection, but the overall lack of an effective *S. aureus* vaccine to date indicates that critical phenotypes and factors are not adequately addressed in current vaccines. For the strategies outlined below, both these and future studies examining alternate parameters will be invaluable resources to refine the search for vaccine candidates.

### Genomics/transcriptomics

Identification of vaccine candidates through the systematic search of the genome and identification of putative antigens, mainly surface-associated proteins, using bioinformatics is referred to as ‘reverse vaccinology’ (Rappuoli, 2000). The progression of this field and its significance to vaccine development against serogroup B *N. meningitidis* and group B *Streptococcus* are detailed in reviews by Serruto & Rappuoli (2006), Serruto *et al.* (2009). This method has a number of advantages compared with previously used methods in that there is no need to grow the pathogen *in vitro* and antigen selection can proceed independent of the abundance of *in vivo* expression and immunogenicity. As a result, many unique antigens can be tested that would have been passed over in conventional studies.

Vaccine candidates identified from a single genome in reverse vaccinology must provide *in vivo* protection against multiple clinical strains in correlative animal models to support transition into clinical studies. An approach, known as comparative genomic hybridization (CGH), uses a DNA microarray of a sequenced ‘reference’ strain to screen for the presence or absence of genes within nonsequenced ‘test’ strains and limits the candidates to antigens conserved in multiple strains. However, the modern ability of advanced sequencing methods such as pyrosequencing has enabled whole-genome sequencing for multiple genomes from various strains of a microbial species to become commonplace. Access to complete genomes of multiple strains for some bacteria makes sequence comparisons among multiple genomes a favorable alternative to CGH because the comparison accounts for all genes within each strain. Earlier CGH studies and more recent deep strain sequencing have led to a description of the ‘pangenome’ in three parts: a ‘core’ genome comprised of genes conserved in all genes, a distributed genome composed of genes not conserved in one or more strains, and a subgroup comprised of novel genes encoded by a single strain (Tettelin *et al.*, 2002, 2005; Shen *et al.*, 2005; Ehrlich *et al.*, 2008). A protective quadrivalent vaccine for *S. aureus* was assembled from surface proteins, IsdA, IsdB, SdrD, and SdrE, after searching eight genomes and evaluating the protective efficacy of multiple candidate antigens in mice (Stranger-Jones *et al.*, 2006).

The increased access to complete genomes of bacteria has led to the ability to develop unique cDNA microarrays for transcriptomic profiling. Evaluation of the bacterial transcriptome under *in vitro* conditions, mimicking environmental stimuli encountered during host infection, detects upregulated genes that may represent virulence factors and vaccine candidates. Transcriptomic analysis is generally restricted to *in vitro* studies, because bacterial RNA is difficult to extract differentially from the infected host tissue.

### Gene expression technologies: positive selection

Other technologies make use of the *in vivo* transcriptional profiles to gather information on the genes involved in virulence, but circumvent the restrictions of RNA extraction and microarray analysis. Three techniques that analyze *in vivo* gene expression and predict promising vaccine candidates are *in vivo* expression technology (IVET), differential fluorescence induction (DFI), and *in vivo* induced antigen technology (IVIAT) (Mahan *et al.*, 1993; Valdivia & Falkow, 1996; Handfield *et al.*, 2000;).

The first report of IVET applied to a Gram-positive species was a study of *S. aureus* by Lowe *et al.* (1998), using a variation known as recombination-based IVET (RIVET). In the RIVET system, random genomic fragments are fused to a promoterless resolvase gene, such as *tnpR*, to construct a genomic library, and a gene cassette comprised of an antibiotic resistance gene flanked by resolvase recognition sequences is incorporated into the bacterial genome. Excision of the antibiotic marker from the bacterial genome, or ‘resolution’, is dependent on the expression of the *ivi* gene-resolvase fusion, and confers antibiotic sensitivity to the bacterium (Angelichio & Camilli, 2002). Lowe *et al.* (1998) assessed 11 mutants for *ivi* genes that were identified from *S. aureus* genomic libraries screened in a murine renal abscess model and defined seven mutants with attenuated virulence compared with wild-type *S. aureus*. DFI is another promoter-trap approach where promoter induction controls the expression of green fluorescent protein, and microorganisms with gene expression can be isolated by fluorescence-activated cell sorting (Valdivia & Falkow, 1996). Finally, the IVIAT system screens *in vitro* expression libraries of a pathogen with convalescent sera following depletion of antibodies specific to that pathogen grown under *in vitro* conditions.

### Gene expression technologies: negative selection

Signature-tagged mutagenesis (STM) identifies the genes required for *in vivo* growth and survival by screening heterogeneous pools of mutants. Each of the mutants has a transposon with a unique oligonucleotide tag randomly incorporated into their genome. After inoculating pools of mutants into a relevant *in vivo* infection model, those mutants that fail to colonize the model can be identified by their unique transposon tag (Hensel *et al.*, 1995). STM screens of *S. aureus* virulence in murine models of bacteremia, abscess, and wound and rabbit endocarditis have been completed, and report that <10% of the mutants were attenuated in all three murine models (Mei *et al.*, 1997; Coulter *et al.*, 1998;).

### Proteomics

Proteomic profiling examines and identifies the spectrum of proteins expressed in bacteria under varying growth conditions using two-dimensional gel electrophoresis (2DGE) and MS. Detection of membrane and cell wall proteins is a limitation of proteomic profiling due to low abundance and solubility constraints that are caused by protein hydrophobicity, transmembrane domains, and an alkaline isoelectric point (Fountoulakis & Takacs, 2001). Because vaccine strategies focus on surface-associated proteins, proteomic analyses yield limited vaccine candidates unless extraction protocols that solubilize membrane proteins or isoelectric focusing performed in the alkaline pH range are used. Reference maps of *S. aureus* Phillips and VISA surface proteomes following lysostaphin extraction have been published, and among these, membrane- and cell wall-associated proteins are promising candidate antigens that can be tested for immunogenicity and/or protective activity (Nandakumar *et al.*, 2005; Gatlin *et al.*, 2006;). Another strategy, considered a ‘new chapter in reverse vaccinology,’ developed concurrently with the cited work of Nandakuman and colleagues, and Gatlin and colleagues examined surface proteins ‘shaved’ from group A *Streptococcus* using trypsin disgestion (Musser, 2006; Rodriguez-Ortega *et al.*, 2006;). Cell surface shaving proteomics has recently established 42 *S. aureus* COL surface proteins that may have potential for vaccine development (Solis *et al.*, 2010).

Serological probing of proteomic samples, known as immunoproteomics, followed by peptide identification using matrix-assisted laser desorption ionization time-of-flight MS is a direct method for defining antigenic proteins. An initial 2DGE immunoproteomic study of *S. aureus* COL identified 15 known and novel proteins that were immunoreactive with patient sera (Vytvytska *et al.*, 2002). Using subtractive proteome analysis, Glowalla and colleagues selected proteins that were immunoreactive with an intravenous immunoglobulin (IVG) preparation and nonreactive with IVG depleted of *S. aureus*-specific opsonizing antibodies and identified three anchorless cell wall proteins that provided partial protection in a mouse sepsis model (Glowalla *et al.*, 2009). These anchorless wall proteins lack a conserved signal peptide or an LPXTG motif, characteristic of most surface-associated proteins, and in some cases, may be consequently omitted from classical reverse vaccinology screens (e.g. vaccine development from genome analysis) (Chhatwal, 2002). Immunoproteomic studies have also evaluated two obstacles to the clinical control and prevention of *S. aureus*, biofilms that potentiate chronic infections and colonization or human carriage (Brady *et al.*, 2006; Holtfreter *et al.*, 2009;). Indeed, most humans possess pre-existing circulating antibodies against major *S. aureus* virulence factors that do not protect against a subsequent challenge by this pathogen. Incomplete protection may be attributed to the transient nature of virulence factor expression during the infection, which requires consideration during the process of vaccine development.

### Antigenomics

Antigenomic screens probe *Escherichia coli* surface-expressed fusions that express randomly fragmented genomic libraries with human sera that are depleted of *E. coli*-specific antibodies. The screens identify a large repertoire of antigenic peptides including those encoded by alternate reading frames (Etz *et al.*, 2002). Indeed, antigenomic studies of *Staphylococcus* and *Streptococcus* found that 24% of antigens were hypothetical proteins or proteins of unknown function from nonannotated reading frames (e.g. alternative reading frame, complementary strand reading frame, nongene matching reading frame), which are categories eliminated from bioinformatics-based vaccine development (Meinke *et al.*, 2005). Antigenomic peptides can be evaluated for widespread *in vivo* expression, or reactivity, via screening with multiple serum samples and conserved expression among multiple bacterial strains (Etz *et al.*, 2002). High-throughput screening methods that circumvent the restrictive in-frame cloning step and peptide insolubility issues that limit peptide repertoire in the bacterial surface expression systems include phage display and ribosome display. However, antigenomic strategies may inadequately define antigenic peptides compared with *in vitro* expression systems, possibly due to protein toxicity and reduced membrane permeation obstructing surface expression and limiting antigen detection.

### Taking into account the mode of growth: biofilm vs. planktonic

The early pioneering work and the continued modern era of biofilm disease discovery by a number of investigators have transformed the field of medical microbiology (Nickel *et al.*, 1985a, b, 1986a, b, 1989; Post *et al.*, 1996; Ehrlich *et al.*, 2002; Erdos *et al.*, 2003; Murphy *et al.*, 2005; Stoodley *et al.*, 2005; Hall-Stoodley *et al.*, 2006; Hiller *et al.*, 2007; Hogg *et al.*, 2007). Because of these studies, the biofilm mode of growth has been recognized as the major mode of infection, with an estimated 80% of all infections caused by biofilms (National Institutes of Health, 1998, 1999). Although extensive studies have been performed on biofilm infections, the resolution of these infection continues to be the surgical removal of the nidus of infection (Shirtliff & Mader, 2000). This surgical removal is necessary because these microbial communities are 50–500 times more resistant to antimicrobial agents than their planktonic and free-floating counterparts (Nickel *et al.*, 1985b; Stewart & Costerton, 2001;). Although the significance of biofilm infections has been recognized as an important mediator of chronic infection and the resulting morbidity and mortality, vaccine studies have often ignored biofilms in discovery and efficacy studies.

For example, recent vaccine development programs for *S. aureus* have tended to focus on testing the ability of target antigens to protect the host from *in vitro* or murine planktonic infection models (Fattom *et al.*, 1996, 2004; McKenney *et al.*, 1998, 1999, 2000; Stranger-Jones *et al.*, 2006; Bubeck Wardenburg & Schneewind, 2008; Lin *et al.*, 2009; Kim *et al.*, 2010). Infections with *S. aureus* may exist in a biofilm mode of growth either during nares carriage or skin infections. Once transmitted to the circulatory system through an epithelial breach, planktonic growth ensues, where upregulation of adherence factors occurs (Beenken *et al.*, 2004). At this point, the invading staphylococci are either removed by the host innate immune response or attach to host extracellular matrix proteins and develop a localized biofilm community. Once this community develops, the proteome of the microorganisms quickly transforms into a biofilm phenotype. Therefore, the planktonic mode of growth that occurs in sepsis may be a transient state. Also, although the host may be vaccinated against planktonic antigens, they may develop a significant memory response only after the secondary foci of biofilm infection has already occurred and the antigenic nature of this pathogen has also significantly changed, thereby detracting from vaccine efficacy.

In the context of biofilm infections, the first question that must be answered when selecting antigen targets is which component of the biofilm should be targeted. Broadly speaking, two alternatives exist: bacterial cells within the biofilm and the biofilm matrix itself. The biofilm matrix may be composed of polysaccharides, protein, or extracellular DNA, in proportions that vary between bacterial genera, species, and strains. As of 2009, the majority of antibiofilm vaccine efforts have been directed toward the biofilm matrix (Schaffer & Lee, 2008). Perhaps the best example of this is the staphylococcal polysaccharide intercellular adhesin (PIA), which is composed of poly-*N*-acetyl-β-1,6-glucosamine (PNAG). The enzymes that catalyze the production of these polysaccharides are encoded for by the genes of the *icaADBC* locus (Joyce *et al.*, 2003). PIA is produced by both *Staphylococcus epidermidis* (McKenney *et al.*, 1998) and *S*. *aureus* (Cramton *et al.*, 1999), and is known to be involved in the adherence of *S*. *epidermidis* to both host tissues (Costa *et al.*, 2009) and inert biomaterials (Olson *et al.*, 2006). PIA/PNAG plays an additional role in immune evasion in both the biofilm and the planktonic mode of growth. The *icaADBC* locus has been detected in clinical *S*. *epidermidis* isolates (Ziebuhr *et al.*, 1997), and its contribution to pathogenesis has been demonstrated in animal models of infection (Rupp *et al.*, 1999). Hence, upon a superficial review, PIA would seem to be an ideal candidate for a vaccine antigen.

In contrast to *S*. *epidermidis*, PIA production is less pronounced in most *S*. *aureus* strains and often observed *in vitro* only under particular conditions, such as anaerobiosis (Cramton *et al.*, 2001) or relatively high (1%) glucose concentrations (Ammendolia *et al.*, 1999). In one study, only 57% of strains that were *icaADBC* positive by PCR analysis (Arciola *et al.*, 2001a) produced a biofilm when cultured *in vitro* (Knobloch *et al.*, 2002), suggesting distinct strain differences in any correlation of PIA and biofilm formation. *In vivo*, analysis of clinical *S*. *aureus* isolates from prosthetic-joint infections, bacteremia (Fowler *et al.*, 2001), catheter-related infections (Arciola *et al.*, 2001a), or from randomly selected clinical isolates (Martin-Lopez *et al.*, 2002) indicates possession of the *ica* locus by the majority of isolates. However, a lack of PIA production was observed in many of these strains *in vitro*. The proportion of *ica*-positive strains among *S. aureus* clinical isolates is thought to vary according to the clinical origin of the isolate and even between infection sites that are both biofilm mediated. For example, the proportion of *icaADBC*-positive *S*. *aureus* strains was higher in orthopedic prosthesis-associated infection (92%) than in catheter-associated infections (63%) (Rohde *et al.*, 2001). Thus, the site and composition of indwelling biomaterials may act as selective factors for strains with different and alternate adhesion mechanisms. The situation is further complicated by the fact that possession by a staphylococcal strain of the *icaADBC* locus does not necessarily mean that PIA will be produced *in vivo*. Similarly, the production of PIA *in vitro* does not mean that it will be produced *in vivo* during an infection. In addition, *in vitro* PIA expression may differ between assays (Rohde *et al.*, 2001). Although there is some evidence that suggests a correlation between *icaADBC* possession and slime production *in vitro* (Arciola *et al.*, 2001b), more research is required to fully understand the importance of PIA in staphylococcal infection *in vivo*. There is also limited evidence that suggests that PIA expression can undergo phase variation (Ziebuhr *et al.*, 1997).

A vaccine based on PIA has undergone trials in animal models. McKenney *et al.* (1998) used PNAG to immunize mice. Five days after an intravenous challenge with two *S*. *aureus* strains (CP5 Reynolds and CP8 MN8), both of which are negative for PNAG production *in vitro*, immunized mice showed a significant reduction in CFU recovered from the kidneys as compared with the controls (McKenney *et al.*, 1999). Further work by the same group suggested that the deacetylated form of PNAG, dPNAG (15% acetylation), conjugated to the diphtheria toxoid is more effective as a vaccine than the 90% acetylated form (Maira-Litran *et al.*, 2005). This is likely due to the retention of dPNAG on the bacterial cell surface, in contrast to the highly acetylated PNAG form, which is released into suspension (Cerca *et al.*, 2007). The deacetylase activity of the *icaB* gene product (Vuong *et al.*, 2004) mediates this effect. The use of PNAG as a vaccine has shown promise in subsequent studies in animal models of *S*. *aureus* mastitis (Perez *et al.*, 2009) and *S*. *aureus* skin abscess (Gening *et al.*, 2010). Given that PNAG is produced by a variety of other bacterial taxa, including *E. coli* (Wang *et al.*, 2004), *Actinobacillus actinomycetemcomitans*, *Actinobacillus pleuropneumoniae* (Kaplan *et al.*, 2004), *Bordetella* spp. (Parise *et al.*, 2007), and *Acinetobacter baumannii* (Choi *et al.*, 2009), PNAG has shown promise in subsequent vaccine studies in animal models of *E*. *coli* bacteremia (Cerca *et al.*, 2007) and peritonitis (Gening *et al.*, 2010).

The efficacy of a PNAG-based vaccine against *S*. *aureus* biofilm-type infection remains to be elucidated. However, given that possession of the *icaADBC* locus by clinically isolated *S*. *aureus* varies between infection sites (Rohde *et al.*, 2001), PNAG may not be the ideal vaccine antigen in a formulation intended to prevent biofilm-type infections. Besides PIA/PNAG, other biofilm factors have simply not been evaluated extensively and these may potentially be inappropriate targets in subsequent studies. Also, one may question whether it would be more efficacious to promote the host immune response to attack the cells producing the matrix or attack the matrix itself. The extracellular matrix of a biofilm community exists, at least in part, to act as an immunoavoidance mechanism. Furthermore, in many cases, the matrix material is constantly being produced and sloughing off into the environment.

## Polymicrobial diseases: considerations for vaccine development

Although many infectious diseases are initiated by a single pathogen or virulence factor, others originate from or are attributed to a complex milieu of microorganisms. Examples of diseases associated with both polymicrobial and biofilm phenotypes include periodontal disease, otitis media, rhinosinusitis, ventilator-associated pneumonia, and chronic wound infections (Brogden *et al.*, 2005). These biofilm consortia of microorganisms typically coexist as combinations of highly structured communities of bacteria, viruses, protozoans, and fungi attached to biotic and environmental surfaces, where their architecture is facilitated by specific intermicrobial and host interactions (Bakaletz, 1995; Viale & Stefani, 2006; Kuramitsu *et al.*, 2007;). Many of these interactions are mutually beneficial for both the host and the microorganism (e.g. the gastrointestinal and oral microbiota). However, microbial species population shifts and waning host immunity can allow colonization and subsequent infection by opportunistic pathogens that exploit unique niches in the polymicrobial environment (Stecher & Hardt, 2008). Despite the challenges of implementing polymicrobial vaccines, several have been attempted and proven successful, while others have yielded unexpected findings.

Traditionally, the guidelines for vaccine development for monomicrobial infections often rely heavily on molecular Koch's postulates, such that directing an immune response against a single virulence or colonization factor will provide protection against disease (Falkow, 1988). Although these rules have proven invaluable for vaccination against several diseases (e.g. *C. diphtheriae*), they do not adequately consider the pathogenesis of polymicrobial infections. It has been well documented that biofilm communities demonstrate a significantly different repertoire of gene and protein expression as compared with their planktonic counterparts (Dykes *et al.*, 2003; Waite *et al.*, 2006;). However, little is known about the transcriptomic and proteomic profiles of multispecies biofilms assessed against monomicrobial communities. The pleiotropic effects of intermicrobial interactions on the individual disease-causing pathogens and the infected host are only now being appreciated. A recent study by Sibley *et al.* (2008) used a *Drosophila* polymicrobial disease model and luciferase reporter assay analyses to examine the effects of human oropharyngeal commensal isolates in coculture with *Pseudomonas aeruginosa* during infection. The results from this study demonstrated that the virulence of *P. aeruginosa* could be substantially enhanced or reduced dependent on the presence of a coinfecting microorganism that was nonpathogenic independently. Even more surprising was the modulation of host antimicrobial and innate immunity genes due specifically to polymicrobial vs. monomicrobial infection. These altered microbial and host profiles are likely due to the unique physical interactions and chemical signaling events that occur during the development of polymicrobial communities (Hogan *et al.*, 2004; Bamford *et al.*, 2009;). Therefore, antigenic targets should be screened *in vivo*, via biologically relevant routes of infection or colonization, to ensure that immunogenic proteins of interest are expressed during infection and in the context of a polymicrobial environment as has been described previously (Rollenhagen *et al.*, 2004; Brady *et al.*, 2006; Hagan & Mobley, 2007;).

The impact of the polymicrobial nature of a disease regarding colonization and infection should also be considered during vaccine development. A disease must first be classified as truly polymicrobial based on sufficient data from clinical studies and epidemiological records. Important criteria regarding the temporal shifts, composition, abundance, and consistency of microorganisms present throughout the entire course of the disease, from colonization to fulminant infection, should be considered (Roberts, 1989; Tarsia *et al.*, 2007;). One must also distinguish contaminating microorganisms (pathogens or commensals) from those that initiate and propagate infection. If a disease is considered to be of a polymicrobial nature, a vaccine composed of a multivalent cocktail of antigenic proteins from all microorganisms involved in disease pathology may be warranted. Although seemingly trivial, these criteria are crucial to understanding the pathogenesis of and developing effective vaccines for multimicrobial diseases.

Polymicrobial infections represent a significant complexity in vaccine development. Two (or more) microorganisms may act synergistically or antagonistically to mediate disease while either in isolation is differentially virulent or benign (Carlson, 1983; Diebel *et al.*, 1999;). Even if a vaccination attempt successfully negates a necessary virulence factor for one pathogen (i.e. a toxin), virulence could be complemented *in trans* by another factor produced by a neighboring species in the polymicrobial community. In addition, the eradication of one species from the polymicrobial community may be insufficient at reducing overall disease, as another organism present may fill in the niche left behind. Alternately, a vaccination attempt targeting a virulence factor (i.e. an adhesin) for one pathogen may successfully target and eradicate a secondary pathogen within the polymicrobial infection.

Modulation of a microorganism's pathogenicity by the polymicrobial community has important implications for vaccine development as studies for *S. aureus* suggest. A formidable nosocomial pathogen, *S. aureus* can be isolated as the single etiologic agent in a multitude of diseases (e.g. sepsis, lower respiratory tract infections, skin infections, and others) or among a polymicrobial community in the same disease types. Polymicrobial infections complicate approximately 27% of nosocomial *Candida albicans* bloodstream infections; among these, *S. aureus* is the third most common coinfecting microorganism (Klotz *et al.*, 2007). As microbial biofilms on indwelling medical devices act as a potential nidus for planktonic release and onset of sepsis, observations of enhanced biofilm formation and differential matrix composition for *S. aureus* in coculture with *C. albicans* suggest that polymicrobial interactions may facilitate *S. aureus* colonization and disease onset (Harriott & Noverr, 2009). The synergistic action of *C. albicans* and *S. aureus* has also been implicated in the increased mortality of mice infected with *S. aureus* strains producing the toxic shock toxin (Carlson, 1983). Indeed, vaccination against *C. albicans* using the candidal adhesion Als3P can provide cross-kingdom protection against *C. albicans* and *S. aureus*, and has positive implications for controlling diseases mediated by coinfection of these microorganisms (Spellberg *et al.*, 2008).

In summary, polymicrobial infections require ecological and physiological characterization to determine interactomes and changes in target expression based on community characteristics. Therefore, vaccine design for polymicrobial infections should adequately consider the consortia of microorganisms responsible for disease, potential intermicrobial interactions resulting in the modulation of *in vivo* expressed antigens, and the strategic elimination of microorganisms that enhance or contribute to pathogenesis. Future strategies may be to target vaccination against seemingly nonpathogenic organisms that facilitate increased pathogenicity and colonization of virulent microorganisms. Of course, vaccination against ‘commensals’ may have deleterious immunological and microbiological consequences in the host and will have to be tested rigorously before utilization.

## Considerations for future vaccines: lessons learned from *S. aureus*

Effective vaccines are available today for many previously problematic bacterial infections, such as the triple vaccine against *C. diphtheriae*, *C. tetani*, *Bordetella pertussis* (Pichichero *et al.*, 2006), *N. meningitidis* (Trotter *et al.*, 2008), and *S. pneumoniae* (Bernatoniene & Finn, 2005). The infections targeted by these vaccines are all mediated by one or a few virulence factors, which, when blocked or otherwise neutralized, prevents pathogenesis. Alternatively, other microorganisms have presented a significant challenge in vaccine development due to a complex disease process and the presence and expression patterns of their respective virulence factors. One such example is *S. aureus*. This pathogenic species is able to cause a host of different types of infections that are either planktonic (e.g. sepsis and pneumonia), biofilm mediated (e.g. osteomyelitis, endocarditis, chronic skin infections, indwelling medical device infections, chronic rhinosinusitis, dental implantitis, and endophthalmitis), or a combination of both modes of growth (e.g. abscess).

*Staphylococcus aureus* is able to accomplish this array of infections by possessing nearly 70 virulence factors, each with infectious mode-of-growth and time-specific expression patterns. Therefore, the search for a single candidate antigen effective in all these cases has hindered *S*. *aureus* vaccine development. Additionally, the ability of these vaccines to provide protection against multiple modes of growth, including both planktonic and biofilm infection, has not been addressed adequately. While the suggestion of a prophylactic vaccine against the biofilm mode of growth seems counterintuitive, details emerging about *S. aureus* pathogenicity and modulation of the host immune response support this concept. In addition to the multitude of innate immunity evasion tactics (e.g. inhibition of neutrophil chemotaxis, inactivation of complement factors, depletion of leukocyte levels, and inhibition of phagocytosis) (Foster, 2005), *in vitro* and *in vivo* studies indicate that *S. aureus* factors direct the host response toward a beneficial one for the pathogen. *In vitro* cytokine analyses demonstrate a robust Th1 immune response elicited against *S. aureus*: staphylococcal enterotoxin B induces IL-2 and IFN-γ (Assenmacher *et al.*, 1998), staphylococcal enterotoxin B induces THF-α and MIP-1β (Dauwalder *et al.*, 2006), and whole-cell *S. aureus* induces IL-12 p70 and IL-18 (Buzas *et al.*, 2004). Studies in a murine model of prosthetic implant infected with *S. aureus* found upregulation of Th1 cytokines (IL-2, IL-12 p70, and TNF-α) and Th17 cytokines (IL-6 and IL-17) at days 7 and 28 postinfection and increased levels of IgG2b (the dominant Th1-dependent iso-subtype) compared with IgG1 (a Th-2 dependent iso-subtype) in the serum at day 7 postinfection (R. Prabhakara & M. E. Shirtliff, unpublished data). These studies indicate that *S. aureus* elicits a prolonged Th1 response, where the proinflammatory defenses are thwarted by the microbial virulence factors and cause significant damage to the host tissue, and subverts a Th2 humoral response; these skewed immune responses allow the planktonic *S. aureus* to elude clearance by the immune system as the microorganism colonizes the damaged host tissue and forms a biofilm. Therefore, in order to encompass all aspects of staphylococcal virulence in vaccine development, one must also include an emphasis on biofilms.

### Antigen selection: the next generation

In order to correctly select appropriate antigens that will be effective in preventing the establishment of a microbial infection, it is necessary to take into account the planktonic and biofilm modes of growth. Microbial biofilms present a unique challenge to researchers seeking to develop vaccines against microorganisms whose infectivity depends, wholly or in part, on this growth modality. Success cannot be achieved by ignoring the fundamental principle of microbial biofilms: *biofilm*-*resident bacterial cells exhibit a phenotype that is distinct*, *and in some cases*, *almost unrecognizable*, *compared with that of taxonomically identical cells growing planktonically* (Beenken *et al.*, 2004; O'May *et al.*, 2009;). Thus, both the planktonic and the biofilm phenotype and its implications for antigen expression must be taken into account during the selection of antigens to be included in a vaccine. While the search for a single antigen that provides multimodal protection may prove successful, it seems more likely that a multicomponent vaccine will be necessary. This is the first criterion for an effective broad-range vaccine.

The second is to ensure that the selected antigens are expressed in all relevant strains of the pathogen targeted by the vaccine. The genetic variation of surface-expressed proteins between strains also raises a difficulty. Just such a problem (Thompson *et al.*, 2003; Dyet & Martin, 2005;) as well as the structural homology of the polysaccharide capsule with the polysialylated form of the neural cell adhesion molecule (Finne *et al.*, 1983) has held up the development of a broad-range vaccine against type B *N. meningitidis*, although clinical trials have begun on vaccines developed by reverse vaccinology and other strategies (Granoff, 2010; Sadarangani & Pollard, 2010;). For this reason, it is vital to test vaccine efficacy against as large a number of strains as is realistically feasible.

The third principle is to ensure that the candidate antigens are expressed *in vivo* throughout the infection cycle in the multiple types of infection (e.g. sepsis vs. indwelling medical device infection) for which the pathogen is the identified etiological agent. Once again, like the multiple modes of growth, this protection will most likely need to be accomplished by a multivalent vaccine.

The fourth principle of antigen selection is that either (1) the selected antigen, or (2) the sum of all antigens included in a multicomponent vaccine, must be expressed throughout the infecting microbial population. This is particularly the case when prevention of biofilm-type infections is the goal. Biofilm communities are inherently complex systems, usually existing in close proximity to a surface. This complexity arises from a number of factors. First, distinct physicochemical gradients are found within microbial biofilm communities. In most cases, organic compounds, oxygen, or water enter the biofilm from the surrounding bulk fluid and diffuse through the matrix to the depths closer to the surface. Bacteria resident within a biofilm consume these compounds at varying rates, resulting in differential availability of nutrients, dependent on the location of a particular cell within the community. This effect has been observed experimentally in the case of oxygen tension (de Beer *et al.*, 1994). The situation is further complicated by very low metabolic levels and radically downregulated rates of cell division of the deeply entrenched microorganisms (Brown *et al.*, 1988), including totally nondividing ‘persister’ cells (Harrison *et al.*, 2005; Lewis, 2008;). This lowered growth rate is partially responsible for the increased recalcitrance to antimicrobials exhibited by biofilm-embedded bacteria (Gilbert *et al.*, 2002). The end result of this is that cells in different areas of the biofilm exhibit spatial phenotypic heterogeneity, i.e. an antigen expressed by cells in a relatively nutrient-rich area of the community may not be expressed by other cells under less favorable growth conditions. A study by Brady *et al.* (2006) on *S*. *aureus* investigated the ability of polyclonal IgG raised in rabbits against antigens, shown in an earlier work by the same authors to be expressed in *S*. *aureus* biofilm *in vivo*, to visualize *S*. *aureus* biofilm communities grown in an *in vitro* flow reactor (Brady *et al.*, 2007). Data suggested that although each of the four antigens was expressed within *S*. *aureus* biofilm communities, none of them was expressed homogenously throughout the biofilm. Instead, differing expression patterns were observed for each antigen. Hence, inclusion of any one antigen in a monovalent vaccine would likely mean that only a fraction of the biofilm would be targeted and the biofilm would likely survive and the infection would persist. It follows that a multivalent vaccine is essential when prevention of biofilm-type infection is the goal.

Finally, the antigens selected for a biofilm vaccine must be immunologically relevant, meaning that they must be cell-surface proteins that are visible to the humoral immune system and not obscured by the biofilm matrix. Furthermore, each component must be capable of not only eliciting a strong humoral immune response in the host, but a correct response. In some cases, microbial clearance can be promoted by either an inflammatory response (Th1 and/or Th17) or an anti-inflammatory response (Th2 and/or Treg) that can be disease mode, species, or even microbial strain specific. Once again, multivalent vaccines seem to be required to accomplish this principle.

Brady and colleagues used these criteria to select four protein antigens that were demonstrably expressed during *S*. *aureus* biofilm growth *in vitro*, cell-surface associated, and immunogenic in the rabbit model of osteomyelitis (Mader & Shirtliff, 1999; Brady *et al.*, 2007;). Singly, combined with the TiterMax™ adjuvant comprised of squalene, sorbitan monooleate 80, and a synthetic block copolymer CRL8941, these antigens were unable to provide protection against *S*. *aureus* osteomyelitis in the rabbit model. However, when used together as a prophylactic quadrivalent vaccine (75 μg of each protein administered subcutaneously; one booster 14 days later; both using the TiterMax™ adjuvant) and combined with postinfection vancomycin treatment (5 mg kg^−1^ twice daily for 10 days) to eliminate planktonic bacteria residing within the bone, eight of nine animals cleared the infection completely. Furthermore, there were significant reductions in radiological and clinical signs of infection in the treated vs. the untreated groups (Brady *et al.*, in press). Research now being conducted is seeking to include *S*. *aureus* surface proteins expressed during planktonic growth in order to remove the need for concurrent vancomycin administration.

The unique physiology and properties of biofilm must be taken into account when selecting antigens for inclusion in any vaccine intended to be effective against these communities. Biofilm-type infections can no longer be regarded as merely ‘bacteria embedded within slime’. Biofilm-resident microorganisms are distinct from their free-living counterparts and present unique challenges to anyone seeking to develop novel prophylactic therapeutics.

## Conclusions

Vaccine development has primarily focused on the pathogenesis of a single microorganism based on its virulence and immunoavoidance factors and the directed host response to the monomicrobial infection. However, greater appreciation of the fact that many infectious diseases result and persist due to the polymicrobial nature and biofilm maturation of bacteria is challenging many perceptions on vaccine design. Current recombinant vaccines targeting a single or a few bacterial proteins possess the benefits of easy manufacture, no risk of disease from reversion back to a virulent form, and few adverse effects from inflammatory induction compared with whole-cell vaccines. Recombinant vaccine usage does come with the loss of antigen diversity and robust humoral response due to the innate response activation that is provided from vaccination with whole cells. As such, redundancy in bacterial proteins expressed during infection, for example adhesins, subverts responses activated by monovalent vaccines and provides incomplete protection. Antigenic variation has also compelled reassessment of vaccine design due to the observation that in vaccinated individuals the diseases targeted by current clinical vaccines, for example *S. pneumoniae* 7-valent, shift toward ones actuated by previously scarce and inconsequential bacterial variants that are not represented in the vaccine (Eskola *et al.*, 2001). Multivalent strategies have come to the forefront in vaccine development in hopes to provide antigenic diversity and sufficient vaccine efficacy, but some clinical trials with multivalent vaccines fail to transition into a later phase, due to the incomplete coverage against disease that is observed.

*Staphylococcus aureus*-mediated diseases highlight the key properties of the pathogen that are challenges to current vaccine strategies and not appropriately addressed during most vaccine development efforts, including polymicrobial infection, biofilm maturation, and host carrier status. Vaccines targeting *S. aureus* adherence factors could be ineffective against diseases where coinfecting microorganisms contribute virulence factors in *trans* and negate the activity of the *S. aureus* factors, for example hypothetical control of *S. aureus* adherence by the *B. pertussis* secreted proteins during coinfection that mimics *in vitro* findings (Tuomanen, 1986). Once *S. aureus* colonization is successful and *S. aureus* immunoavoidance factors obstruct the innate immune response, *S. aureus* may grow and persist as a biofilm community encapsulated in a polysaccharide matrix. Compounding the problem is that this timed up- and downregulated expression of virulence factors is not only growth phase dependent but also disease specific.

The biofilm phenotype further conceals *S. aureus* from the immune system due to the downregulated expression of factors that mediate initial infection and encapsulation in polysaccharide that masks surface-associated proteins from immune recognition. Analysis of the mature *S. aureus* biofilm indicates that there is great heterogeneity in protein expression throughout the biofilm community, with protein expression present in some microcolonies and completely absent in others. As such, a vaccine that targeted these proteins would be ineffective at eliciting an opsonization response to clear *S. aureus*.

Another consideration for vaccine development is the expression of virulence factors that antagonize the immune response, inducing inflammation and tissue damage, where further bacterial colonization can occur; other factors that target and inactivate host immunoglobulins also pose significant problems. Knowledge of the specific immune responses activated by the bacteria and whether that response assists bacterial colonization and persistence will allow the development of vaccines that can modulate the immune response, using adjuvants or extrinsic bacterial components, which skew toward appropriate immunity.

A final consideration for vaccine development is *S. aureus* carriage in humans. Analysis of sera from healthy carriers establishes the circulation of anti-*S. aureus* immunoglobulins, indicating that this response is insufficient to prevent colonization and persistence. Vaccine strategies using antigens targeted by those immunoglobulins will probably elicit a response that is not completely protective. Therefore, screening for and removal of those antigens before protection studies may be advisable. Overall, these properties are critical to understanding how the immune response is ineffective at bacterial clearance. Further evaluation of these features will establish optimal antigenic candidates, including protein factors specific for disease and those not concealed from the immune system that should be established as prerequisites for *S. aureus* and other bacterial vaccines.

## Statement

Re-use of this article is permitted in accordance with the Terms and Conditions set out at http://www3.interscience.wiley.com/authorresources/onlineopen.html
